# TimeTraits: extracting functional traits from biological time-series in R

**DOI:** 10.1093/bioadv/vbag226

**Published:** 2026-08-11

**Authors:** Sarah C L Lock, Marina I Knight, Seth J Davis, Daphne Ezer

**Affiliations:** Department of Biology, University of York, Heslington, York YO10 5DD, United Kingdom; Department of Biology, University of York, Heslington, York YO10 5DD, United Kingdom; Department of Biology, University of York, Heslington, York YO10 5DD, United Kingdom; Department of Biology, University of York, Heslington, York YO10 5DD, United Kingdom

## Abstract

**Motivation:**

It is currently challenging for scientists to extract meaningful parameters from circadian rhythms that dynamically change over time (i.e. non-stationary rhythms). Circadian rhythms may be non-stationary due to dynamic conditions, such as changes in photoperiod. Circadian rhythms are also nonstationary when plants are suddenly placed under continuous light conditions after a period of entrainment, as their clocks slowly drift without external entrainment signals.

**Results:**

TimeTraits provides a series of functions to enable the extraction of parameters from time series data using Functional Data Analysis methods. We demonstrate its utility in dissecting the changes in curve shape of a circadian clock bioluminescence marker, following a photoperiod shift in wildtype and phyB Arabidopsis.

**Availability and implementation:**

TimeTraits is available in CRAN: https://cran.r-project.org/web/packages/TimeTraits

## 1 Introduction

Circadian rhythms govern essential processes in animals and plants, including sleep-wake cycles, photosynthesis and growth. For animals, understanding circadian rhythms is crucial for timing medical interventions (Dibner and Schibler 2015, Ayyar and Sukumaran 2021). In plants, they offer insights into optimising agricultural strategies and enhancing resilience to environmental stressors (Minoli *et al.* 2022, Ficht *et al.* 2023, Ullah *et al.* 2024, Schmidt *et al.* 2025). Therefore, how these rhythms are analysed is very important.

Traditionally, the Fast Fourier Transform and Non-linear Least Squares (FFT-NLLS) approach has been widely employed for analysing bioluminescence circadian data (Moore *et al.* 2014, Gould *et al.* 2018, Brancaccio *et al.* 2019). However, this method assumes that the underlying rhythms are stationary (i.e. that the rhythms are consistent over the time series). This assumption potentially leads to oversimplified interpretations and limiting the ability of scientists to investigate rhythms under environmental conditions that change over time. It has been shown that expression of core Arabidopsis thaliana clock genes over time do not look like typical sine-waves and are in fact non-stationary (Hargreaves *et al.* 2019). Alongside this, often biologists summarise properties of the circadian clock in terms of three key features: amplitude, phase, period. By exclusively investigating these features, information about how the gene expression curves deviate from sine-waves are overlooked.

Functional Data Analysis (FDA) statistical methods consider the entire profile of gene expression over time, without many assumptions about which features are important (Ramsay *et al.* 2009). This approach offers a more nuanced and flexible framework for analysing time-dependent data, particularly suitable for circadian data analysis. Viewing data as functions rather than as discrete points enables the exploration of dynamic patterns. For example, FDA methods can highlight subtle changes in waveform happening over time. This approach gives the ability to uncover hidden patterns and complexities that might be overlooked by conventional methods. Furthermore, FDA techniques offer powerful tools for functional regression, clustering, and classification, allowing for the integration of covariates and the identification of distinct temporal patterns within datasets (Ramsay *et al.* 2009, 2024). This capability is particularly valuable in circadian research, where external factors such as light exposure, genetic variations, or environmental conditions can influence rhythmic patterns. By incorporating these additional features into the analysis, FDA facilitates a more thorough investigation of the underlying mechanisms governing circadian rhythms and their interactions with external stimuli.

The adoption of FDA approaches for circadian data analysis has yet to be extensively explored within the biology community, despite the availability of several R packages implementing FDA methodologies, including fda, refund and RespirAnalyzer (Goldsmith *et al.* 2016, Ramsay *et al.* 2024, Zhang *et al.* 2024). These packages provide powerful statistical frameworks for smoothing, functional principal component analysis (FPCA), and functional regression, but often require substantial data wrangling, statistical expertise, and integration across multiple tools. In parallel, circadian focused packages such as MetaCycle primarily concentrate on traditional rhythmicity metrics including period, phase, and amplitude, rather than the full functional structure of curves (Wu *et al.* 2016). A comparison of existing packages and the functionality provided by TimeTraits is summarised in Table 1. In addition, a comparison of the analytical workflows and outputs generated by TimeTraits and a traditional FFT-NLLS pipeline is provided in Fig. S1.

**Table 1:** A comparison of available R packages.

Package	FDA smoothing	FPCA	Circadian-specific	Integrated preprocessing	Outlier detection	Accessiable workflow
fda	Yes	Yes	No	No	No	Moderate
fdaspace	Yes	Yes	No	No	No	Modderate
refund	Yes	Yes	No	No	No	Advanced
MetaCycle	No	No	Yes	Limited	No	Yes
RespirAnalyzer	Limited	Limited	No	Domain-specific	No	Moderate
TimeTraits	Yes	Yes	Yes	Yes	Yes	Yes

The analysis workflow associated with FDA methods also involves multiple preprocessing and modelling decisions, including detrending, basis selection, smoothing parameter estimation, derivative evaluation, and outlier detection, which may present a barrier for researchers without specialist training in functional statistics. To address this gap and promote the utilisation of FDA techniques within the circadian community, we developed TimeTraits, an R package specifically tailored for biological rhythm analyses. Rather than introducing a new FDA methodology, the package integrates established FDA approaches into a unified and accessible workflow that combines preprocessing, curve estimation, FPCA, derivative analysis, outlier detection, and visualisation. By providing user-friendly implementations of these steps, TimeTraits aims to lower the barriers associated with FDA analyses and facilitate exploration of the dynamic and non-stationary nature of circadian rhythms.

## 2 Methods

The ‘package’ has been published at the Comprehensive R Archive Network (CRAN). It is designed to aid the exploration of circadian luminescence data, by providing the user with a collection of prespecified analysis steps thus reducing the complexity of applying FDA methods. The package offers three core functions (i) filter_curves: for pre-processing data directly from bioluminescence lab hardware and performing data quality controls and (ii) smooth_fun: for turning the rhythmic data into normalised functions and (optionally) filtering potential outlier curves. (iii) functional_traits: for extracting a set of non-stationary properties of the rhythms using FDA approaches.

The package conducts three main functions for analysis, starting with preprocessing of the data. The provided R function filter_curves aims to filter circadian luminescent data based on specified criteria, preparing it for further analysis. Initially, the function validates the input data is in the correct format which is a numeric time x sample matrix. Next a data frame containing curves within the specified time range (from to to) is created along with the corresponding time vector. Only curves exceeding the minimum luminescence threshold (min_lum) are identified and retained (Fig. S2).

Many researchers use luciferase assays to produce rhythmic bioluminescent readings, which have signals that decay over time. Moreover, some organisms may die over the course of the experiment and therefore lose their rhythmicity (Creux and Harmer 2019, Paajanen *et al.* 2021). The filter_curves function offers optional methods for de-trending the curves and filtering out samples that lose their rhythmicity in the last 48 hours (or an alternative value set by the user). Detrending is achieved in this example by fitting a linear regression to each circadian rhythm over time and using the residuals to remove any trend (Fig. S3). For datasets exhibiting more complex low-frequency trends or repeated baseline fluctuations, an alternative detrending method is available using moving-average approach. A Lomb-Scargle periodogram analysis is performed for the last 48 hours of the analysis to identify weakly rhythmic curves (Fig. S4) (Ruf 1999). Curves failing to meet the significance threshold (0.001) in the periodogram analysis are removed from the dataset. Finally, the filtered curves are combined back with the corresponding time vector into a final data frame, which is returned as the output of the function, ready for further analysis.

The next function the package provides to the user is aimed to estimate the discrete curves into smooth continuous curves using FDA methods (smooth_fun). Initially, the function checks if the input data is correctly formatted, typically this input will be the preprocessing output. Subsequently, each luminescent curve is normalized to the range [0, 1]. The function then proceeds to create a list containing each curve along with its corresponding time values, removing any missing data points. For each curve, basis functions and functional parameters are generated using FDA methods (Ramsay *et al.* 2024). This involves defining a basis function via B-splines and setting a penalty for roughness that controls the smoothness of the fitted function. The user may define these parameters manually or they can be optimally selected using the helper function select_basis_lambda_LOOCV. This function performs leave-one-out cross-validation across a grid of basis numbers and smoothing parameters (λ), identifying the combination that minimises error (Fig. S5). smooth_fun then applies smoothing to each curve individually (using the chosen basis number and λ), producing smooth basis functions. Next, a new time vector is created to cover the range of all original time values. The curves are evaluated under this new time vector, resulting in a matrix of all curves under the same time points. Outliers in this dataframe are detected using FDA outlier detection methods (‘fdaoutlier’), specifically the Total Variation (TV) Smoothing and Derivative-based Measures of Scale (MSS) (Febrero–Bande and Fuente 2012). Detected outliers are then optionally removed from the dataframe. Finally, the function returns the smoothed curves across the desired time frame providing users with functional data objects ready for further analysis.

The final function in this package (functional_traits), performs FPCA on smoothed circadian time-series data and extracts group-level traits from the resulting FPCA score space. The analysis can optionally be split by time segments (e.g. pre/post environmental shift) and by derivatives of the curves. When segments are supplied, functional observations from all segments are first re-evaluated onto a shared time grid and analysed jointly, ensuring that FPCA scores are directly comparable across segments. For each derivative, FPCA is performed once across all samples and segments simultaneously. Segment- and group-specific traits are then computed post hoc from the shared FPCA score space. The traits are outputted in a table compatible with further R/qtl analysis. Traits calculated include mean and standard deviation of FPCA scores (per principal component), mean and standard deviation of distance to the FPCA-space centroid and convex hull area in PC1–PC2 space (measure of within-group diversity). These traits summarise variation in waveform structure across groups. For example, shifts in mean PC scores indicate systematic differences in overall curve shape between genotypes or treatments, while increased dispersion or convex hull area reflects greater heterogeneity in rhythmic responses within a group. Such traits can be used for downstream statistical analyses, including genotype comparisons, clustering, or quantitative trait locus mapping.

## 3 Example

Traditionally, many circadian experiments are performed under constant conditions to quantify free-running rhythms. However, experiments involving environmental transitions or zeitgeber shifts during continuous recording have been more challenging to analyse using conventional approaches. Here we show that our approach enables us to investigate rhythms under environmental conditions that change over time, specifically having shifts in day lengths in the middle of the experiment, which could be applied to investigate how plants respond to the change in seasons (Ronald *et al.* 2024, Lock *et al.* 2025, Mehta *et al.* 2025). The package comes with this data set manually compiled (phyb). It contains samples of luminescence data from Arabadopsis thaliana seedlings from Ws-2 (harbouring CCR2: LUC) and then Ws-2 plants containing a mutation in phytochromeB gene (phyB-10, also harbouring CCR2: LUC).

**Figure 1:**
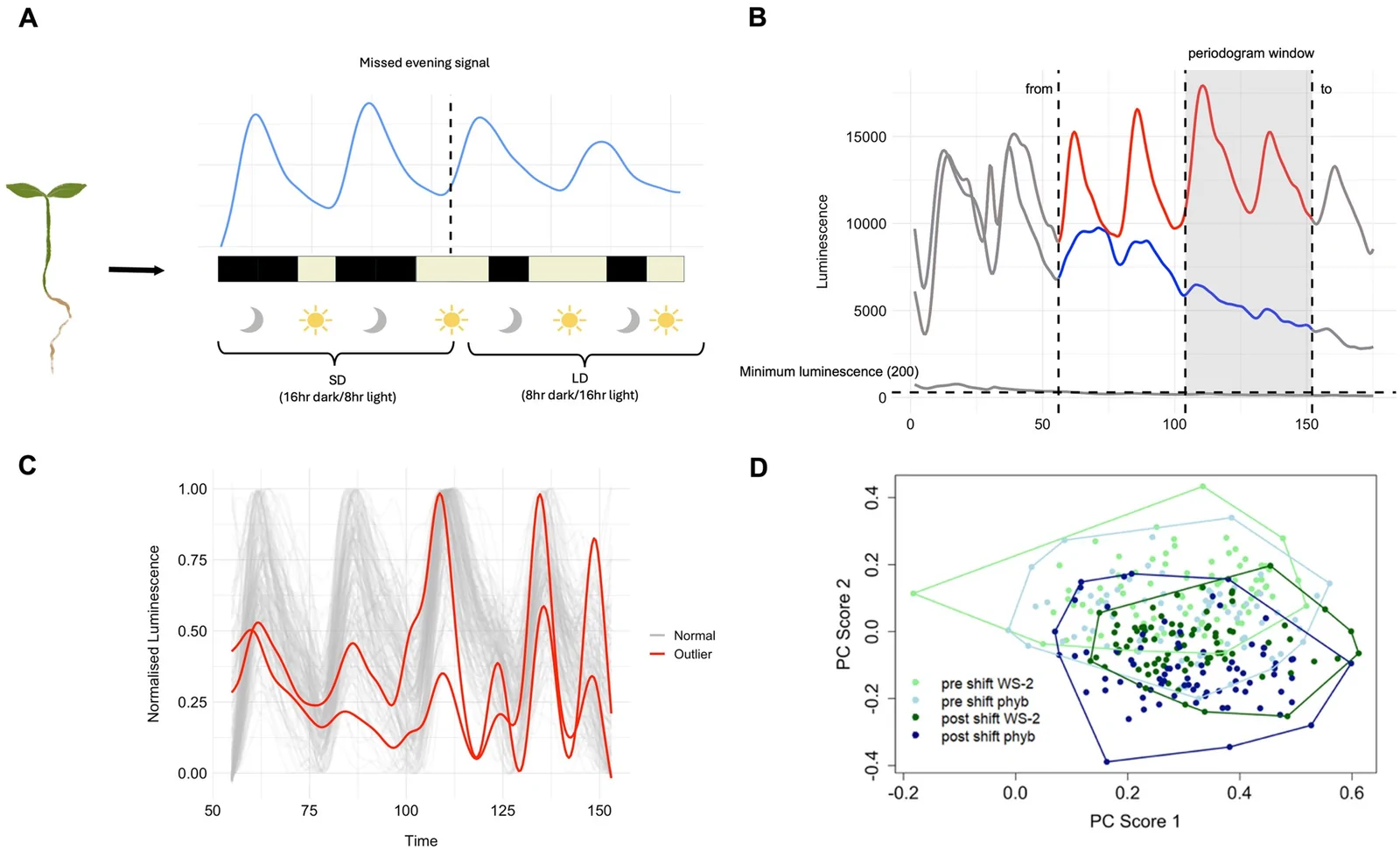
Overview of the TimeTraits analysis pipeline using circadian luciferase data. (A) Experimental design. Arabidopsis thaliana seedlings (Ws-2 wild type and phyB mutants) expressing a luciferase reporter were entrained under a short day (SD, 8 hours light: 16 hours dark) and subsequently shifted to a long day (LD, 16 hours light: 8 hours dark) while luminescence of CCR2: LUC reporter gene was continuously recorded. A dashed black line labelled ‘Missed evening signal’ indicates when the plants would have expected dusk to happen under short day entrainment but due to the shift to long day conditions this was missed. (B) Illustration of curve filtering using the filter_curves function. Shown are the user-defined time window (‘from’, ‘to’), minimum luminescence threshold, and the 48-hour periodogram window (shaded grey) used to assess rhythmicity. Grey curves indicate traces removed during filtering. The blue curve highlights an example rejected due to lack of rhythmicity in the selected periodogram window, while the red curve represents a trace that passed both the minimum luminescence and rhythmicity criteria. (C) Normalised and smoothed luminescence profiles produced by the smooth_fun function. Grey curves represent retained samples, while red curves indicate two of the outliers removed based on overall shape characteristics. (D) Functional principal component analysis (FPCA) of smoothed curves, showing PC1 versus PC2 scores for pre and post photoperiod shift segments across genotypes. Convex hulls summarise group-level structure in FPCA space.

Seeds were surface-sterilized and plated onto MS medium with 3% sucrose and then stratified for 4 days. After stratification, seedlings were entrained under a short day (SD, 8 hours light: 16 hours dark) photoperiod of cycles with a constant temperature of 22° for 7 days. On day 6, seedlings were transferred to black 96-well Microplates with MS medium containing 3% sucrose. Plants were superficially treated with 15 μl 5 mM D-Luciferin. Seedlings were then re-entrained for 1 day under the respective entrainment conditions before being transferred to the TOPCOUNT (Perkin-Elmer [Perkin-Elmer-Cetus], Norwalk, CT) where luminescence measurements began and were taken approximately every 30 minutes over the following 6 days. On day 11 seedlings were then shifted into a long day (LD, 16 hours light: 8 hours dark) photoperiod (see Fig. 1A). TOPCOUNT experiments were carried out under blue-red light and a constant temperature of 21°. The dataset is provided in wide format, with one column per individual seedling either (Ws-2, n = 96 or phyb, n = 96) and one row per time point (hours), consisting of 300 time points.

The filter_curves and the smooth_fun functions were used to perform filtering for unsuitable rhythms and further analysis. The minimum threshold was set at 200 and looking at a window of 44–152 hours and a 48-hour periodogram window, Fig. 1B shows examples of curves that would be included and excluded under these selection parameters. Clear smooth functions were outputted after the removal of outlier curves based on shape (Fig. 1C). After acquiring smooth curves these plants were then evaluated as pre and post photoperiod shift and a FPCA applied using functional_traits (Fig. 1D). Our analysis shows that our methods effectively filter arrhythmic genes, remove outlier curves, and produce smooth normalised curves. These smoothed curves were then used for downstream FDA-based analyses, including FPCA and exploration of waveform variation following the photoperiod shift (Fig. 1D). A key advantage of FDA is that it allows direct analysis of the full functional structure of the data through the original curves, the first derivative (rate of change), and the second derivative (rate of change of the rate of change, i.e. curvature). Unlike FFT-NLLS approaches, which primarily summarise rhythmic behaviour using parameters such as period, phase, and amplitude, FDA provides a more flexible framework for capturing dynamic features of the entire curves, including differences that emerge in the underlying derivatives of the signal. From these functional representations, a series of interpretable FPCA derived traits were extracted to quantify dominant waveform structure, treatment/genotype separation, within-group synchrony, and population heterogeneity (Table 2).

**Table 2:** Example of traits, the interpretation and example biological meaning when using the functional_traits function.

Trait	Interpretation	Example biological meaning
PC1 mean	Dominant waveform	Dominant response to particular condition (i.e light cue)
PC2	Secondary waveform variation	Secondary response, such as curve shift, indicating genotype separation
PC1_dist_centriod	Within group consistency	Consistency of rhythmic responses within population
PC1_PC2_hull_area	FPCA space dispersion	Degree of population heterogeneity
1^st^ derivative PC scores	Rate of change dynamics	Speed/timing of response to photoperiod shift
2^nd^ derivative scores	Acceleration/curvature dynamics	Abruptness or smoothness of transitions

To demonstrate biological interpretation of the extracted FPCA traits, the first derivative FPCA scores were examined, these represents the rate of change over time (Table S1). Separation between pre- and post-photoperiod shift samples was most apparent along PC2 (Fig. S6), indicating changes in curve dynamics following photoperiod shift. Significant effects of both genotype (F_1,318_ = 47.1, *P* = 3.5 × 10^−11^) and photoperiod shift position (F_1,318_ = 185.6, *P* < 2.2 × 10^−16^) were identified for first derivative PC2 scores, while the interaction term was not significant. After the photoperiod shift, phyb mutants showed significantly lower PC2 scores relative to WS-2 plants (*P* = 1.7 × 10^−5^), suggesting altered response in heterogeny over time. These observations are consistent with previous research demonstrating the role of phyB in regulating photoperiodic responsiveness and adaptation to photoperiod shifts (Ronald *et al.* 2024). FFT-NLLS analysis detected a significant increase in circadian period following the photoperiod shift in both WS-2 (paired t-test, *P* = 5.17 × 10^−6^) and phyB (paired t-test, *P* = 2.03 × 10^−8^) plants but identified no significant differences between genotypes either before or after the shift (Fig. S7, Table S2).

## 4 Conclusion

Although demonstrated here using high-resolution circadian luciferase data, the FDA framework implemented in TimeTraits is broadly applicable to other time series biological datasets for example, leaf movement data, chlorophyll fluorescence and gene expression data. B-spline basis functions, as used here, are usually able to effectively fit non-sinusoidal and non-stationary rhythms with fewer degrees of freedom than Fourier basis functions, as used in FFT-based methods (Ramsay *et al.* 2024). As FDA treats observations as continuous functions rather than discrete stationary signals, the approach is well suited.

The smoothing procedures implemented in TimeTraits within smooth_fun can accommodate unevenly spaced observations, provided sufficient coverage exists to estimate reliable functional curves. However, datasets with very sparse sampling or extremely short durations may reduce the stability of spline estimation and downstream FPCA analyses. In such cases, careful selection of smoothing parameters and biological interpretation are required.

In addition to analysing environmental perturbation experiments, the framework may also be useful for genotype screening applications under constant conditions. Even in the absence of external entrainment cues, such as continuous light, phase, period and amplitude of circadian oscillations can exhibit drift over time (Hargreaves *et al.* 2019). Traditional FFT-NLLS approaches assume that these rhythmic parameters remain stationary throughout the analysed time window and therefore may fail to capture such non-stationary features. By treating observations as continuous functions, FDA enables analysis of changes in waveform structures and dynamics across the entire experiment even in screens under constant conditions, providing a complimentary approach for identifying genotypic differences that may not be fully captured by conventional rhythmicity metrics alone.

In addition to analysing environmental perturbation experiments, the framework may also be useful for genotype screening applications under constant conditions. Even in the absence of external entrainment cues, such as continuous light, phase, period and amplitude of circadian oscillations can exhibit drift over time (Hargreaves *et al.* 2019). Traditional FFT-NLLS approaches assume that these rhythmic parameters remain stationary throughout the analysed time window and therefore may fail to capture such non-stationary features. By treating observations as continuous functions, FDA enables analysis of changes in waveform structures and dynamics across the entire experiment even in screens under constant conditions, providing a complimentary approach for identifying genotypic differences that may not be fully captured by conventional rhythmicity metrics alone.

In conclusion this package successfully achieves its aim of providing an accessible and automated workflow for analysing biological time series data using FDA approaches. By integrating the processing, filtering, detrending, outlier removal and functional smoothing into a single framework it reduces the complexity of applying FDA methods. Compared with traditional approaches to analyse circadian data which assume stationarity and focus on a small number of rhythmic parameters this package captures the full functional profile of circadian curves enabling the exploration of non-stationarity and response to external cues over time. This advancement broadens the analytical possibilities for circadian researchers, supporting biologically meaningful exploration and interpretations of rhythmic data.

## Supplementary Material

vbag226_Supplementary_Data

## Data Availability

TimeTraits is an open-source CRAN R package. Source code and example datasets used in this study are available at https://github.com/scllock/TimeTraits.

## References

[vbag226-B1] Ayyar VS , Sukumaran S. Circadian rhythms: influence on physiology, pharmacology, and therapeutic interventions. J Pharmacokinet Pharmacodyn 2021;48:321–38. 33797011 10.1007/s10928-021-09751-2 PMC8015932

[vbag226-B2] Brancaccio M , Edwards MD, Patton AP et al Cell-autonomous clock of astrocytes drives circadian behavior in mammals. Science 2019;363:187–92. 30630934 10.1126/science.aat4104 PMC6440650

[vbag226-B3] Creux N , Harmer S. Circadian rhythms in plants. Cold Spring Harb Perspect Biol 2019;11:a034611. 31138544 10.1101/cshperspect.a034611 PMC6719598

[vbag226-B4] Dibner C , Schibler U. Circadian timing of metabolism in animal models and humans. J Intern Med 2015;277:513–27. 25599827 10.1111/joim.12347

[vbag226-B5] Febrero–Bande M , Fuente MDL. Statistical computing in functional data analysis: the R package fda.Usc. J Stat Softw 2012;051:1–28.

[vbag226-B6] Ficht A , Bruch A, Rajcan I et al Evaluation of the impact of photoperiod and light intensity on decreasing days to maturity in winter wheat. Crop Sci 2023;63:812–21.

[vbag226-B7] Goldsmith J et al 2016. Refund: regression with functional data. R package version 0.1-16.

[vbag226-B8] Gould PD , Domijan M, Greenwood M et al Coordination of robust single cell rhythms in the Arabidopsis circadian clock via spatial waves of gene expression. eLife 2018;7:e31700. 29697372 10.7554/eLife.31700 PMC5988422

[vbag226-B9] Hargreaves JK , Knight MI, Pitchford JW et al Wavelet spectral testing: application to nonstationary circadian rhythms. Ann Appl Stat 2019;13:1817–46.

[vbag226-B10] Lock SCL et al 2025. The genetic basis for synchronised time perception in plant populations. bioRxiv, 2025.02.25.640030.

[vbag226-B11] Mehta D et al 2025. The plant circadian clock exerts stronger control over the diel proteome than the transcriptome. bioRxiv, 2025.12.19.695194.

[vbag226-B12] Minoli S , Jägermeyr J, Asseng S et al Global crop yields can be lifted by timely adaptation of growing periods to climate change. Nat Commun 2022;13:7079. 36400762 10.1038/s41467-022-34411-5 PMC9674574

[vbag226-B13] Moore A , Zielinski T, Millar AJ et al Online period estimation and determination of rhythmicity in circadian data, using the BioDare data infrastructure. Methods Mol Biol 2014;1158:13–44. 24792042 10.1007/978-1-4939-0700-7_2

[vbag226-B14] Paajanen P , Lane de Barros Dantas L, Dodd AN et al Layers of crosstalk between circadian regulation and environmental signalling in plants. Curr Biol 2021;31:R399–413. 33905701 10.1016/j.cub.2021.03.046

[vbag226-B15] Ramsay JO et al Introduction to functional data analysis. In: Functional Data Analysis with R and MATLAB. New York: Springer, 2009, 1–19.

[vbag226-B16] Ramsay JO et al 2024. Package ‘fda’.

[vbag226-B17] Ronald J et al 2024. Hypocotyl development in Arabidopsis and other Brassicaceae displays evidence of photoperiodic memory. bioRxiv, 2024.05.13.593876.

[vbag226-B18] Ruf T. The Lomb-Scargle periodogram in biological rhythm research: analysis of incomplete and unequally spaced time-series. Biol. Rhythm Res 1999;30:178–201. 10.1076/brhm.30.2.149.1424 11708361

[vbag226-B19] Schmidt C , Hinterberger V, Philipp N et al Hybrid grain production in wheat benefits from synchronized flowering and high female flower receptivity. J Exp Bot 2025;76:445–60. 39441002 10.1093/jxb/erae430

[vbag226-B20] Ullah N , Collins B, Christopher JT et al Pre- and post-flowering impacts of natural heatwaves on yield components in wheat. Field Crops Res 2024;316:109489.

[vbag226-B22] Wu G , Anafi RC, Hughes ME et al MetaCycle: an integrated R package to evaluate periodicity in large scale data. Bioinformatics 2016;32:3351–3. 27378304 10.1093/bioinformatics/btw405 PMC5079475

[vbag226-B23] Zhang T , Dong X, Wang D et al RespirAnalyzer: an R package for analyzing data from continuous monitoring of respiratory signals. Bioinform Adv 2024;4:vbae003. 38269257 10.1093/bioadv/vbae003 PMC10807906

