# Supplementary material for: TimeTraits: extracting functional traits from biological time-series in R
Source: Bioinform Adv. 2026 Aug 11;6(1):vbag226. doi: 10.1093/bioadv/vbag226 (PMC13537318; doi:10.1093/bioadv/vbag226)
Supplement: Supplementary file 1 [file vbag226_supplementary_data.zip › Supplementary Information.docx]

**Supplementary Information**

**Supplementary Figure 1:** Comparison of the TimeTraits FDA workflow and a traditional FFT-NLLS circadian analysis pipeline.

**Supplementary Figure 2:** Shows the number of curves removed within each genotype when increasing minimum luminescence threshold (min_lum) when using filter_curves function.

**Supplementary Figure 3:** Shows the effect of detrending the data when using filter_curves in two different example curves.

**Supplementary Figure 4:** Shows the effect of changing the length of the periodogram during filtering using filter_curves.

**Supplementary Figure 5:** Algorithm for picking number of basis functions and smoothing penalty using select_basis_lambda_LOOCV. (A – D) Shows an increase in basis functions decrease cross validation (CV) error. This reaches a plateau where more added basis functions does not improve fit and can lead to overfitting problems.

**Supplementary Figure 6:** Violin plots show the density distribution of first derivative FPCA PC2 scores for Ws-2 wild-type (WS) and phyB-10 mutant (phyb) seedlings before (pre) and after (post) a photoperiod transition from short to long-day conditions. Boxplots indicate the median and interquartile range, while individual points represent scores from individual seedlings. Positive and negative shifts in PC2 scores reflect differences in luminescence profile structure associated with genotype and photoperiod transition.

**Supplementary Figure 7:** Individual estimates off circadian period using FFT-NLLS implemented in Biodare2 (ref) for pre shift (56-104 h) and post shift (104-152h) for Ws-2 and phyB genotypes. Each point represents a single plant and paired observations are connected by lines. Thick lines indicate the genotype mean at each time point. Circadian period increased significantly following the photoperiod shift in both Ws-2 (paired t-test, p = 5.17 x 10-6) and phyB (paired t-test, p = 2.03 x 10-8). No significant differences in circadian period were detected between genotypes either before or after the photoperiod shift.

**Supplementary Method 1:**

The smooth_fun function represents each time series as a smooth functional curve using cubic B-spline basis functions. For a given observed time series $y_{i}(t)$, the smoothed function is estimated as:

$$y_{i}(t)=\sum_{k=1}^{K} c_{ik}\phi_{k}(t)$$

where $\phi_{k}(t)$are cubic B-spline basis functions and $c_{ik}$are basis coefficients estimated from the data.

Smoothing is performed using penalised least squares with a second-derivative roughness penalty:

$$\sum_{j=1}^{n} (y_{ij}-x_{i}(t_{j}))^{2}+\lambda\int\left( D^{2}x_{i}(t) \right)^{2}dt$$

where $\lambda$ controls the trade-off between goodness-of-fit and smoothness, and $D^{2}x_{i}(t)$denotes the second derivative of the fitted function. Larger $\lambda$ values produce smoother curves.
